# Altered microstate C and D dynamics in high social anxiety: a resting-state EEG study

**DOI:** 10.3389/fpsyg.2025.1581517

**Published:** 2025-05-08

**Authors:** Huoyin Zhang, Binyu Peng, Yutong Liu, Yu Xi, Yi Lei

**Affiliations:** ^1^School of Psychology, Shenzhen University, Shenzhen, China; ^2^Institute of Brain and Psychological Science, Sichuan Normal University, Chengdu, China; ^3^College of Psychology, Sichuan Normal University, Chengdu, China

**Keywords:** social anxiety, EEG microstates, resting-state EEG, microstate C, microstate D

## Abstract

**Introduction:**

Social anxiety is characterized by excessive fear of negative evaluation and avoidance in social situations. While its neural processing patterns are well-documented, the millisecond-level temporal dynamics of brain functional networks remain poorly understood. This study used EEG microstate analysis to explore the dynamic neural mechanisms underlying social anxiety.

**Methods:**

Eyes-closed resting-state EEG data were collected from 41 participants, divided into high social anxiety (*n* = 23) and low social anxiety (*n* = 18) groups based on their Liebowitz Social Anxiety Scale (LSAS) scores. EEG microstate parameters, including duration, occurrence frequency, time coverage, and transition probabilities, were analyzed. Correlation analyses were conducted between LSAS scores and microstate dynamics.

**Results:**

The high social anxiety group exhibited significantly increased duration and coverage of microstate C (associated with processing personally significant information and self-reflection) and decreased duration and coverage of microstate D (associated with executive functioning). Transition probabilities involving microstate C (A ↔ C, B ↔ C) were significantly higher, while those involving microstate D (A ↔ D) were significantly lower in the high social anxiety group. In the low social anxiety group, B ↔ C transition probability showed significant negative correlations with LSAS total and avoidance subscale scores.

**Discussion:**

These findings reveal distinct neural dynamics in social anxiety, characterized by heightened self-referential processing (microstate C) and impaired executive functioning (microstate D). The altered transition patterns suggest a predisposition for excessive self-focus and reduced coordination with executive control networks in high social anxiety individuals. These results provide new insights into the neural mechanisms of social anxiety and offer potential directions for clinical interventions and early detection.

## 1 Introduction

Social anxiety is a common psychological trait characterized by excessive fear of negative evaluation and avoidance tendencies in social situations (Leary, 1983; Rapee and Spence, 2004). A recent meta-analysis by Tang et al. (2022) revealed a 23.5% prevalence of social anxiety symptoms in China, with substantial impacts on academic, social, and professional functioning. Examining the dynamic characteristics of brain functional networks in individuals with high social anxiety traits may provide novel temporal evidence for understanding the underlying neural mechanisms.

Social anxiety demonstrates distinct characteristics from other anxiety types, such as generalized anxiety. Specifically, social anxiety is characterized by fear and avoidance of social situations, with its core features being excessive attention to social evaluation and negative anticipation, manifesting as intense fear of both negative and positive evaluation (Clark and Wells, 1995; Morrison et al., 2024). In contrast, generalized anxiety manifests as excessive and persistent worry across multiple life domains (Baik and Newman, 2025). Individuals with social anxiety exhibit significant attentional bias in processing self-relevant information and external social cues, showing preferential allocation of attentional resources to self-relevant information (Hou et al., 2024) and atypical multisensory integration patterns when processing social cues such as face-voice combinations (Gan and Li, 2023). These cognitive processing characteristics reinforce their fear and avoidance tendencies in social situations.

Resting-state functional magnetic resonance imaging (fMRI) studies have revealed specific neural circuit alterations in social anxiety. Research has identified functional abnormalities in multiple large-scale brain networks in individuals with high social anxiety traits, primarily manifesting as altered functional connectivity among the default mode network (DMN), subcortical network, and perceptual systems (sensorimotor, auditory, and visual networks) (Zhang et al., 2023). Notably, during resting state, reduced functional connectivity between the DMN and attention and perception networks has been observed (Zhang et al., 2024). In contrast, individuals with generalized anxiety disorder primarily exhibit amygdala hyperactivity and abnormal amygdala-frontal cortex functional connectivity (Qiao et al., 2017), reflecting general alterations in threat monitoring and emotion regulation. These differences in neural mechanisms suggest unique brain network dynamics in social anxiety. However, the temporal resolution limitations of fMRI preclude the detection of millisecond-level neural activity changes, making it impossible to reveal rapid brain functional network state transitions in individuals with high social anxiety traits.

To address the temporal resolution limitations of fMRI, researchers have turned to electroencephalography (EEG) microstate analysis. This method captures stable patterns of brain electrical activity within tens to hundreds of milliseconds, precisely characterizing rapid brain state transitions (Valt et al., 2024). Research has demonstrated that different microstate classes correspond to specific cognitive functions and neural processes: Microstate A is associated with both auditory and visual processing and relates to arousal levels, Microstate B is linked to self-related visual processing and autobiographical memory, while Microstates C and D correspond to self-referential processing/personally significant information processing and executive functioning, respectively (Tarailis et al., 2024). Recent systematic reviews have further clarified that additional microstates (E-G) may represent other functional networks, such as the salience network (E), aspects of the default mode network (F), and the somatosensory network (G) (Tarailis et al., 2024). This functional mapping, while not strictly one-to-one (Britz et al., 2010; Musso et al., 2010), provides crucial evidence for understanding the temporal dynamics of cognitive processes. EEG microstate analysis offers a unique perspective by assessing both temporal characteristics (frequency, duration, and coverage) and transition patterns between different microstates.

Using this analytical approach, research on emotional disorders has accumulated several important findings. Regarding temporal characteristics, patients with generalized anxiety disorder show reduced functionality in D-class microstates, manifested as decreased duration, occurrence, and coverage, along with reduced transition probabilities from other states to microstate D and increased transitions between states C and E (Hao et al., 2025). Research on depressive symptoms found that while there were no between-group differences in temporal characteristics, symptom severity positively correlated with the occurrence of microstate A (Damborská et al., 2019). Recent research has revealed that anxiety symptoms were significantly correlated with microstate E parameters (coverage and occurrence) and transitions from microstate B to E, with these parameters showing superior predictive power for anxiety symptoms (Xue et al., 2024). However, the specific microstate characteristics of social anxiety, a common but distinct form of anxiety disorder, remain largely unexplored. Given its unique features of fear and avoidance in social situations, investigating the microstate patterns in social anxiety could provide valuable insights into its neural mechanisms.

Compared to temporal characteristics, research on microstate transition patterns has opened new perspectives for understanding anxiety’s neural mechanisms. Specifically, the microstate transition probability matrix reflects preferential patterns of transitions between different functional states, serving as a crucial indicator of brain information processing flexibility (Khanna et al., 2015). Studies of emotional disorders have found that participants show decreased B ↔ D transition probabilities, while A → D and B → C transition probabilities increase (Al Zoubi et al., 2019), suggesting abnormal dynamic switching of brain functional networks in emotional disorders. Similarly, Xue et al. (2024) reported that anxiety symptoms were associated with specific transition patterns, particularly transitions from microstate B to E (B → E), further supporting the importance of examining network transition dynamics in understanding anxiety symptoms.

While these studies have revealed general characteristics of anxiety, current microstate research has primarily focused on generalized anxiety or mixed anxiety samples, with relatively few studies examining EEG microstate transition characteristics in social anxiety. Given that individuals with social anxiety exhibit specific cognitive processing biases and emotion regulation difficulties in social situations, particularly considering their unique cognitive processing features such as excessive self-focus and social threat monitoring (Rapee and Heimberg, 1997; Clark and Wells, 1995), their microstate dynamic characteristics may present specific patterns. This may be particularly evident in brain network states involving personally significant information processing and self-referential internal mentation (Microstate C) and executive functioning (Microstate D), potentially showing dynamic characteristics distinct from other types of anxiety. Therefore, investigating millisecond-level neural dynamics and transition efficiency holds unique value for understanding the cognitive neural basis of social anxiety and may provide new insights into its neural mechanisms.

In addition, existing studies suggest that there may be a direct correlation between brain network dynamic features and the severity of clinical symptoms (Damborská et al., 2019; Xue et al., 2024). For example, in depression, the activity of microstate A was positively correlated with the severity of depressive symptoms (Damborská et al., 2019), whereas anxiety symptoms were significantly correlated with the coverage of microstate E (Xue et al., 2024). These findings suggest that correlation analyses combining behavioral indicators (e.g., Social Anxiety Scale scores) with neurodynamic parameters may provide important clues to understanding individual differences in social anxiety. Therefore, the present study will further explore the association between the severity of social anxiety symptoms and microstate dynamic features (e.g., duration, coverage).

While brain functional network dynamics are closely related to the development of social anxiety traits, current understanding of its neural mechanisms remains largely limited to static functional connectivity. The present study collected resting-state EEG data and employed microstate analysis to examine differences in microstate temporal characteristics and transition patterns between high and low social anxiety groups. Based on previous findings of cognitive biases in social anxiety (Hou et al., 2024; Gan and Li, 2023) and abnormal microstate dynamics in emotional disorders (Al Zoubi et al., 2019), we hypothesize that:(1) The high social anxiety trait group will show altered temporal characteristics (e.g., duration, frequency) in Microstate C (associated with processing personally significant information and self-referential internal mentation) and Microstate D (associated with executive functioning) compared to the low social anxiety group; (2) The high social anxiety trait group will exhibit different transition probabilities involving Microstate C and Microstate D compared to the low social anxiety group. (3) The temporal characteristics and transition patterns of Microstates C and D will be significantly correlated with social anxiety symptom severity scores. By examining the dynamic characteristics of brain functional networks in individuals with high social anxiety traits, this study provides temporal dynamic evidence for the neural mechanisms of social anxiety while offering objective neurophysiological indicators for clinical assessment and early intervention.

## 2 Methodology

### 2.1 Participants

A total of 1858 social anxiety questionnaires were distributed via the Naodao platform (Chen et al., 2023) and social media, yielding 1,445 valid responses (78% validity, with a median of 62). Initially, 45 college students were recruited, of which 4 participants were excluded due to excessive head movement or excessive EEG artifacts, resulting in a final sample of 41 valid participants. Based on the scores of the Liebowitz Social Anxiety Scale (LSAS).We divided the high and low social anxiety groups by a median of 62 calculated from a large sample (Carol et al., 1982), the participants were divided into a high social anxiety group (*n* = 23, 19 females) and a low social anxiety group (*n* = 18, 13 females). Independent samples *t*-tests showed significant differences between the two groups in LSAS total score [*t*_(39)_ = 13.791, *p* < 0.001, Cohen’s *d* = 4.338], fear dimension [*t*_(39)_ = 11.804, *p* < 0.001, Cohen’s *d* = 3.716], and avoidance dimension [*t*_(39)_ = 8.750, *p* < 0.001, Cohen’s *d* = 2.756], indicating effective grouping (see Table 1).

**Table 1:** Participant demographics information.

Variant	High social anxiety (*M ± SD*)	Low social anxiety (*M ± SD*)	*x*^2^/*t*(39)	*p*
Sex (Male/Female)	4/19	5/13	0.425	/
Age	19.870 ± 1.324	19.280 ± 1.018	0.125	0.493
LSAS	82.170 ± 12.702	27.830 ± 12.282	13.791***	<0.001
LSAS-Fear/Anxiety	41.610 ± 8.419	13.110 ± 6.579	11.804***	<0.001
LSAS-Avoidance	40.570 ± 9.811	14.720 ± 8.804	8.750***	<0.001

LSAS = Liebowitz Social Anxiety Scale; M: mean; SD: standard deviation.*p < 0.05, **p < 0.01, ***p < 0.001.

All participants were right-handed, had normal or corrected-to-normal vision, and self-reported no history of mental illness. All participants signed a written informed consent form before participating in the experiment and received monetary compensation. This study was approved by the Ethics Committee of Sichuan Normal University (approval number: SCNU-211120, approval date: November 20, 2021), and the experimental process strictly followed the Declaration of Helsinki. In this study, the Cronbach’s *α* coefficient for the LSAS scale (containing 24 social situations, each requiring a rating of fear and avoidance on a scale of 0–3) was 0.954, with 0.961 for the fear/anxiety subscale and 0.973 for the avoidance subscale, indicating good internal consistency of the scale (Liebowitz, 1987; He and Zhang, 2004).

### 2.2 Experimental procedure

This study employed a resting-state EEG paradigm. The experiment was divided into three stages: preparation, data acquisition, and conclusion. During the preparation stage, participants first completed a demographic information survey and the Chinese version of the LSAS scale through the Questionnaire Star platform.

The experiment was conducted in a soundproof and softly lit EEG laboratory. EEG data were collected using a 64-channel EEG amplifier system (Brain Products, Germany) with a sampling rate of 1000Hz and a bandpass filter of 0.1-100Hz. The reference electrodes were placed on bilateral mastoids, and the ground electrode was placed on the forehead. The impedance of all electrodes was maintained below 5kΩ. Resting-state EEG data were recorded with a quick cap carrying 64 Ag/AgCl electrodes placed at standard locations covering the whole scalp (the extended international 10–20 system) (Li et al., 2022).Participants maintained a distance of 60 cm from the monitor and performed two task conditions: 5 min of eyes-closed resting state and 5 min of eyes-open resting state (fixating on a central fixation point “+” on the screen). Before each task, standardized instructions were presented to the participants, requiring them to remain relaxed but alert and to avoid thinking about specific issues. A rest period of no less than 2 min was set between the two task conditions. During the experiment, the experimenter monitored the participants’ status in real-time through a monitor and, when necessary, reminded them to stay awake through an intercom system.

After the experiment, participants rested for a moment, removed the electrode cap, and had their scalp appropriately cleaned. Consistent with previous works, only data collected under an eyes-closed state were analyzed.

### 2.3 EEG preprocessing

EEG data were pre-processed using EEGLAB 13 (Delorme and Makeig, 2004), an open source toolbox running in the MATLAB 2013b environment, and in-house MATLAB functions. First, the data were bandpass filtered at 0.5–80 Hz and notch filtered at 50 Hz to remove high-frequency noise and power line interference. Then the raw data were segmented into continuous 2 s epochs. When there were artifacts in a channel, the spherical interpolation method was used for interpolation, and <6 channels for each subject were replaced. When the signal quality of a segment was poor (the voltages of more than 10 channels exceed 80 μV), it would be excluded. After that, each subject retained at least 3 min EEG signals. Eye artifact correction was performed using independent component analysis (Jung et al., 2000). Specifically, we corrected EEG signal segments contaminated by eye movements, blinks, electromyographic (EMG) disturbances, electrocardiographic (ECG) artifacts, or non-physiological noises by means of the Infomax independent component analysis (ICA) algorithm. Second, independent components associated with artifacts were manually identified according to the morphological criteria developed by Chaumon et al. (2015). Data were then re-referenced to the average reference. During EEG data preprocessing, for the high social anxiety group, the numbers of interpolated channels and deleted epochs (mean ± SD) were 4.2 ± 1.3 and 7.8 ± 4.7 respectively, with 142.5 ± 5.2 remaining epochs. For the low social anxiety group, the numbers were 4.4 ± 1.5 and 8.2 ± 5.0 for interpolated channels and deleted epochs respectively, with 141.5 ± 4.5 remaining epochs.

### 2.4 Microstate analysis

Consistent with recent work (Damborská et al., 2019; Valt et al., 2024), we performed microstate analysis using Cartool software. In to published studies, 2–20 Hz band filtering was used for digital filtering (Kim et al., 2021; Li et al., 2022), for this reason we digitally filtered the EEG data after preprocessing through a 2–20 Hz bandpass filter. Then, the software first computed the global field power (GFP) to capture the instantaneous changes in the EEG signal. Since the topographic maps around GFP peaks are the most stable and representative of instantaneous activity from a signal-to-noise ratio perspective, voltage topographic maps at these time points were extracted for subsequent analysis (Pascual-Marqui et al., 1995). We then estimated the number of clusters at the individual level according to the criteria implemented in Cartool (i.e., Davies and Bouldin, Gamma, Silhouette, Dunn Robust, Point-Biserial, Krzanowski-Lai index, and cross-validation). Four clusters emerged as the optimal solution across these criteria. The topographic maps occurring at the peaks of the GFP curve were entered into a Topographic Atomize and Agglomerate Hierarchical Clustering (T-AAHC) algorithm to isolate four microstate clusters within each dataset (Brunet et al., 2011). This result is also consistent with the number of microstate classes widely accepted in traditional classic studies (Michel and Koenig, 2018). The topographic maps occurring at the peaks of the GFP curve were entered into a Topographic Atomize and Agglomerate Hierarchical Clustering (T-AAHC) algorithm to isolate four microstate clusters within each dataset. Temporal Agglomerative Hierarchical Clustering (T-AAHC) employs a bottom-up approach, which reduces the number of clusters gradually from an initial large set compared to the K-means clustering algorithm, thus significantly reducing the computational effort (Brunet et al., 2011). Therefore, the T-AAHC method was used in this study. In addition, previous neuroimaging studies have successfully used T-AAHC in a similar analytical setting (Jia et al., 2024; Brodbeck et al., 2012). This result is also consistent with the number of microstate classes widely accepted in traditional classic studies (Michel and Koenig, 2018). Each sampling point was assigned to the corresponding microstate class based on the global map dissimilarity (GMD) metric. To reduce the influence of noise, a 30 ms time window (Besag factor = 10) was used for smoothing, and microstate segments with durations less than 30 ms were removed.

A series of spatiotemporal parameters were calculated for each microstate to assess its dynamic characteristics. These parameters included: mean duration (ms), reflecting the average time that each microstate class remained stable within a unit of time; occurrence frequency (Hz), indicating the average number of times each microstate class occurred within a unit of time; time coverage (%), representing the percentage of total time each microstate class accounted for within a unit of time; and transition probability, the percentage of transitions between different microstates within a unit of time out of the total number of transitions. In addition, global explained variance was calculated to evaluate the overall explanatory power of the microstate model for the EEG signal (Koenig et al., 2002; Michel and Koenig, 2018).

### 2.5 Statistical analysis

Statistical analyses were performed in SPSS 27.0 (IBM Corp., Armonk, NY, United States). First, an independent samples t-test was conducted on the global explained variance of the microstate topographic maps to compare the differences between the high and low social anxiety groups. Second, independent samples *t*-tests were performed to compare microstate parameters (duration, occurrence frequency, time coverage) between high and low social anxiety groups for each microstate class (A/B/C/D) separately. Third, independent samples t-tests were conducted to compare transition probabilities between high and low social anxiety groups for each transition type (A → B, A → C, A → D, B → A, B → C, B → D, C → A, C → B, C → D, D → A, D → B, D → C) separately (Jiang and Zheng, 2024). Additionally, to address the relationship between social anxiety and microstate characteristics, Pearson correlation analyses were conducted between LSAS total scores and subscale scores (fear and avoidance) and microstate parameters (duration, occurrence frequency, time coverage, and transition probabilities between different microstate classes) in both high and low social anxiety groups.

To control for multiple comparisons, Bonferroni correction was applied. For microstate parameters, *p* < 0.013 (i.e., 0.05/4, as there were 4 different types of microstates) was considered statistically significant. For transition probabilities, *p* < 0.004 (i.e., 0.05/12, as there were 12 different types of transitions) was considered statistically significant. Effect sizes were reported using Cohen’s *d*.

## 3 Results

### 3.1 Microstate topographic map characteristics

**Figure 1:**
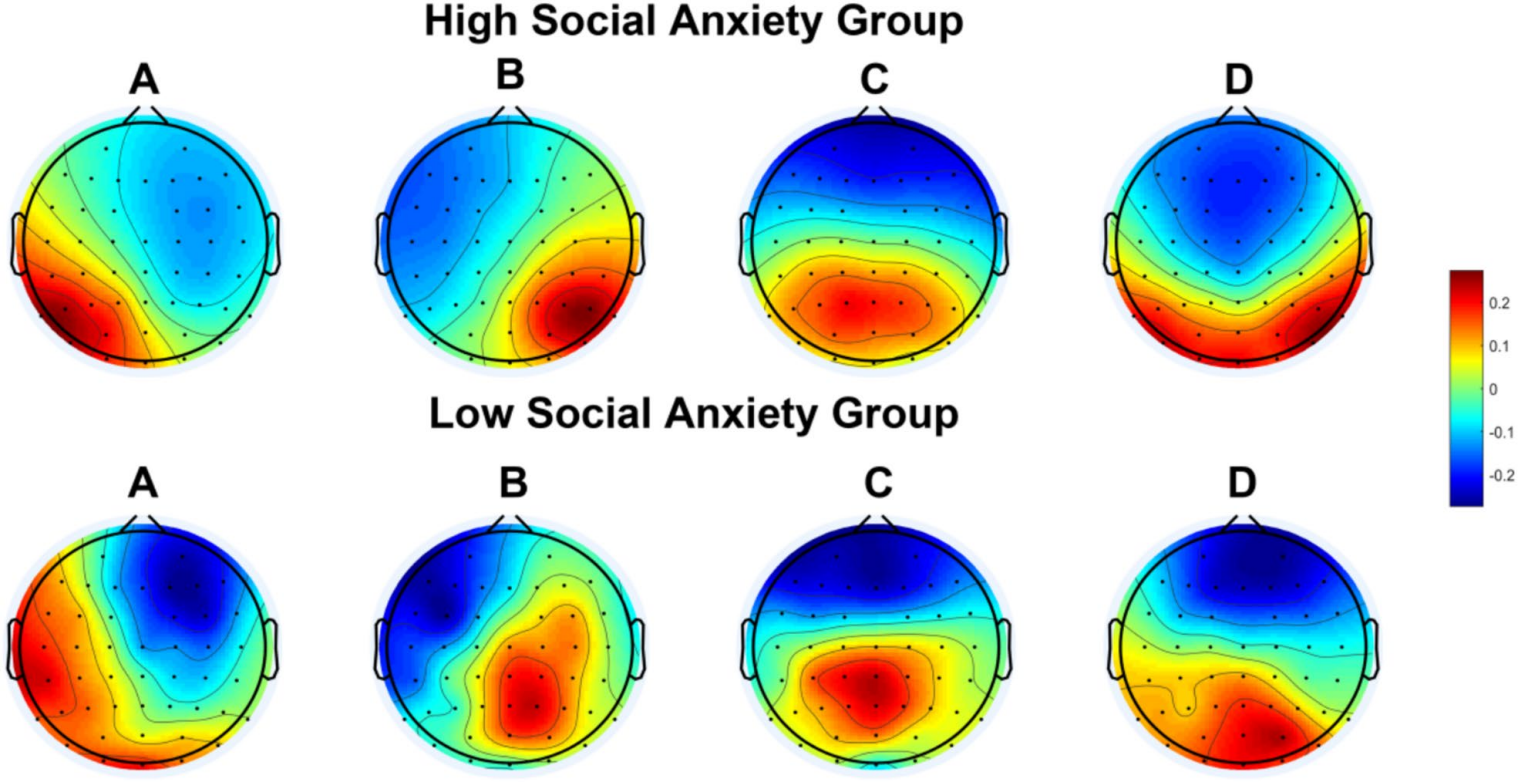
Spatial configuration of the four microstate classes. **(A)** Microstate A; **(B)** microstate B; **(C)** microstate C; **(D)** microstate D. Blue regions are negative and red are positive relative to average reference. The top row shows the EEG topoplots for the high social anxiety group, and the row under it shows the EEG topoplots for the low social anxiety group.

The topographies of the four microstate classes are displayed in Figure 1, both the high and low social anxiety groups exhibited four types of microstate topographic maps. Microstates A, B, and D showed dipole distributions in the left–right, left–right, and anterior–posterior directions, respectively, while class C showed a maximum value distribution in the anterior central region. Among these four microstates, the global explained variance of the high social anxiety group (67.74 ± 5.94%) was significantly higher than that of the low social anxiety group (58.22 ± 7.79%) [*t*_(39)_ = 4.623, *p* < 0.001, Cohen’s *d* = 1.402].

### 3.2 Microstate parameters characteristics

**Figure 2:**
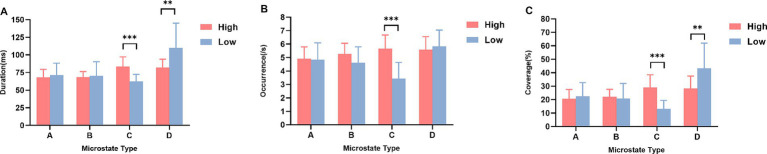
The four microstates parameters between high and low social anxiety groups (M ± SD). **(A)** Duration, **(B)** Occurrence, and **(C)** Coverage for microstates A–D in the high and low social anxiety groups. High: high social anxiety group; Low: low social anxiety group. **p < 0.01, ***p < 0.001.

First, for microstate duration, (1) microstate class C was significantly higher in the high social anxiety group than in the low social anxiety group [*t*_(39)_ = 5.576, *p* < 0.001, Cohen’s *d* = 1.721]; (2) microstate class D was significantly lower in the high social anxiety group than in the low social anxiety group [*t*_(39)_ = −3.189, *p* = 0.005, Cohen’s *d* = −1.087] (Figure 2A). Other effects were not significant (all *p* > 0.013) (Table 2).

**Table 2:** Microstate parameters of high social anxiety group and low social anxiety group.

Microstate Type and Index	**A**		**B**		**C**		**D**	
	Mean	SD	Mean	SD	Mean	SD	Mean	SD
Duration (ms)	69.410	14.170	69.250	14.430	74.240	16.150	94.080	28.430
High	67.810	11.770	68.500	8.080	83.640	13.560	81.880	12.190
Low	71.450	16.890	70.210	20.080	62.230	10.170	109.670	35.360
*t*_(39)_	−0.812		−0.373		5.576		−3.189	
*p*	0.421		0.711		**< 0.001**		**0.005**	
Cohen’s *d*	−0.251		−0.115		1.721		−1.087	
Occurrence (/s)	4.890	1.040	4.980	1.020	4.690	1.560	5.710	1.060
High	4.920	0.870	5.280	0.790	5.670	1.010	5.600	0.950
Low	4.850	1.250	4.610	1.180	3.440	1.210	5.840	1.200
*t*_(39)_	0.226		2.142		6.426		−0.708	
*p*	0.822		0.038		**< 0.001**		0.483	
Cohen’s *d*	−0.221		0.129		1.913		−1.050	
Coverage (%)	21.400	8.510	21.550	8.390	22.120	11.340	34.940	15.850
High	20.550	7.010	22.040	5.640	29.100	9.370	28.310	9.240
Low	22.480	10.230	20.920	11.120	13.200	6.230	43.400	18.590
*t*_(39)_	−0.717		0.419		6.199		−3.152	
*p*	0.478		0.678		**<0.001**		**0.004**	
Cohen’s *d*	0.070		0.661		1.983		−1.028	

Duration (ms): duration of microstates; Occurrence (/s): frequency of microstates per unit of time; Coverage (%): proportion of microstates covered; High: high social anxiety group; Low: low social anxiety group. All data that are statistically significant (*p* < 0.013) are bolded.

Second, for microstate occurrence rate, microstate class C was significantly higher in the high social anxiety group than in the low social anxiety group [*t*_(39)_ = 6.426, *p* < 0.001, Cohen’s *d* = 1.913] (Figure 2B). Other effects were not significant (all *p* > 0.013) (Table 2).

Finally, for microstate time coverage, (1) microstate class C was significantly higher in the high social anxiety group than in the low social anxiety group [*t*_(39)_ = 6.199, *p* < 0.001, Cohen’s *d* = 1.983]; (2) microstate class D was significantly lower in the high social anxiety group than in the low social anxiety group [*t*_(39)_ = −3.152, *p* = 0.004, Cohen’s *d* = −1.028] (Figure 2C). Other effects were not significant (all *p* > 0.013) (Table 2).

### 3.3 Microstate transition characteristics

**Figure 3:**
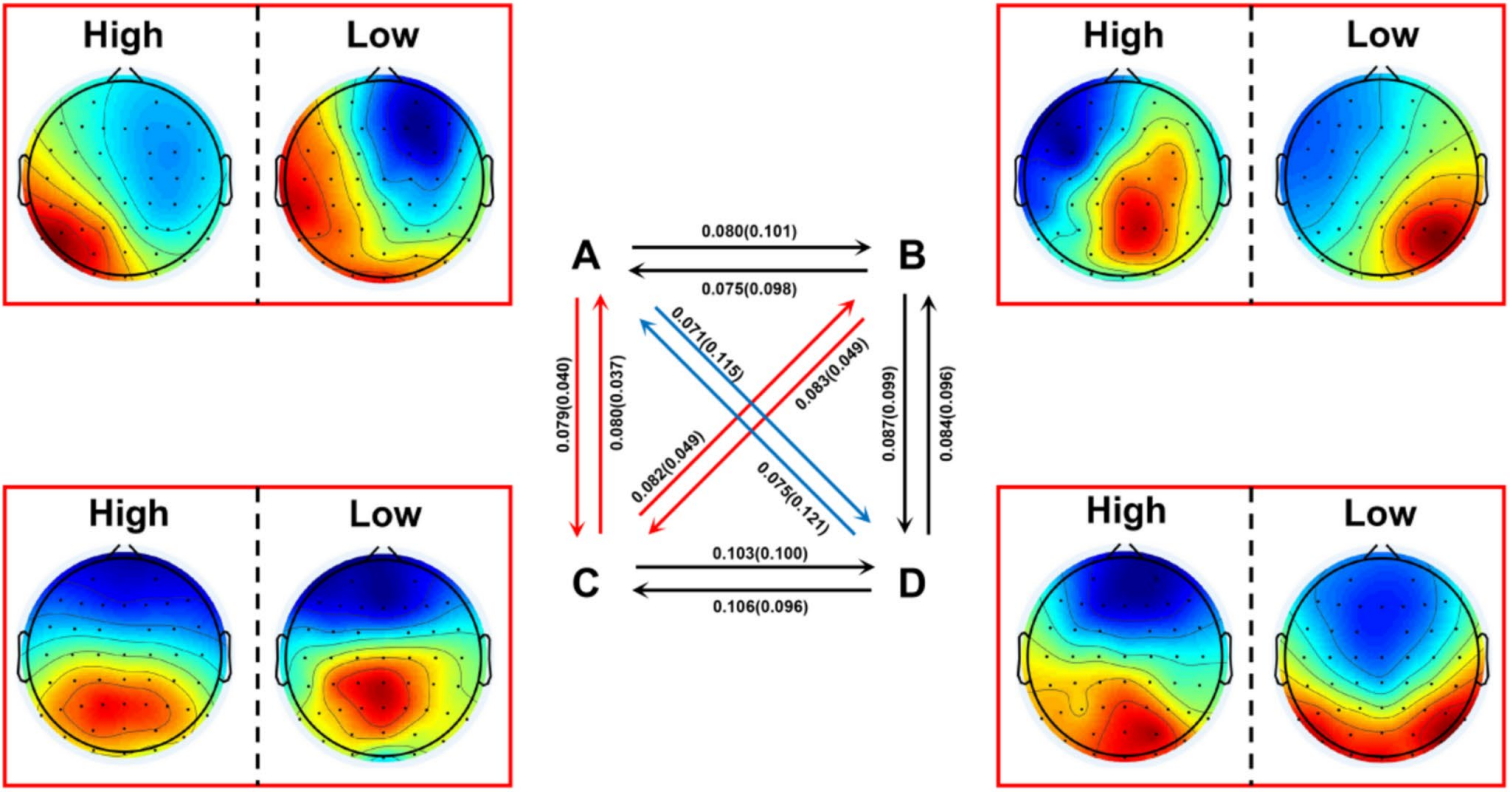
Transition probabilities between the four microstates in the high social anxiety group and low social anxiety group. Red arrows indicate significantly higher transition probabilities in the high social anxiety group (*p* < 0.001); blue arrows indicate significantly higher transition probabilities in the low social anxiety group (*p* < 0.001). The values in brackets above the arrows represent the mean transition probabilities of the high and low social anxiety groups, respectively. High: high social anxiety group; Low: low social anxiety group.

The results showed that the high social anxiety group had significantly higher transition probabilities in A → C [*t*_(39)_ = 4.958, *p* < 0.001, Cohen’s *d* = 1.530], B → C [*t*_(39)_ = 4.774, *p* < 0.001, Cohen’s *d* = 1.473], C → A [*t*_(39)_ = 5.105, *p* < 0.001, Cohen’s *d* = 1.575], and C → B [*t*_(39)_ = 4.770, *p* < 0.001, Cohen’s *d* = 1.472] compared to the low social anxiety group, while the low social anxiety group had significantly higher transition probabilities in A → D [*t*_(39)_ = −5.536, *p* < 0.001, Cohen’s *d* = −1.708] and D → A [*t_(39)_* = −5.267, *p* < 0.001, Cohen’s *d* = −1.625] compared to the high social anxiety group (Figure 3). Other effects were not significant (all *p* > 0.004) (Table 3).

**Table 3:** Comparison and means for all transition probabilities in high and low social anxiety groups.

Transition	High (*n* = 23)	Low (*n* = 18)	*t*_(39)_	*p*	Cohen’s *d*
A to B	0.080 ± 0.015	0.100 ± 0.047	−1.805	0.086	−0.617
**A to C**	**0.079 ± 0.028**	**0.040 ± 0.019**	**4.958**	**< 0.001**	**1.530**
**A to D**	**0.071 ± 0.025**	**0.115 ± 0.026**	**−5.536**	**< 0.001**	**−1.708**
B to A	0.075 ± 0.018	0.097 ± 0.052	−1.740	0.097	−0.594
**B to C**	**0.049 ± 0.020**	**0.087 ± 0.035**	**4.774**	**< 0.001**	**1.473**
B to D	0.087 ± 0.035	0.099 ± 0.043	−0.955	0.345	−0.295
**C to A**	**0.079 ± 0.031**	**0.037 ± 0.019**	**5.105**	**< 0.001**	**1.575**
**C to B**	**0.082 ± 0.023**	**0.049 ± 0.021**	**4.770**	**< 0.001**	**1.472**
C to D	0.103 ± 0.023	0.100 ± 0.070	0.183	0.857	0.063
**D to A**	**0.075 ± 0.025**	**0.121 ± 0.032**	**−5.267**	**< 0.001**	**−1.625**
D to B	0.084 ± 0.028	0.096 ± 0.040	−1.130	0.265	−0.349
D to C	0.102 ± 0.021	0.096 ± 0.065	0.346	0.733	0.118

A to B refers to the transition probability from microstate A to microstate B. The same applies to other expressions; All data that are statistically significant (*p* < 0.004) are bolded.

### 3.4 Correlation between microstate parameters and transitions with social anxiety scale

**Figure 4:**
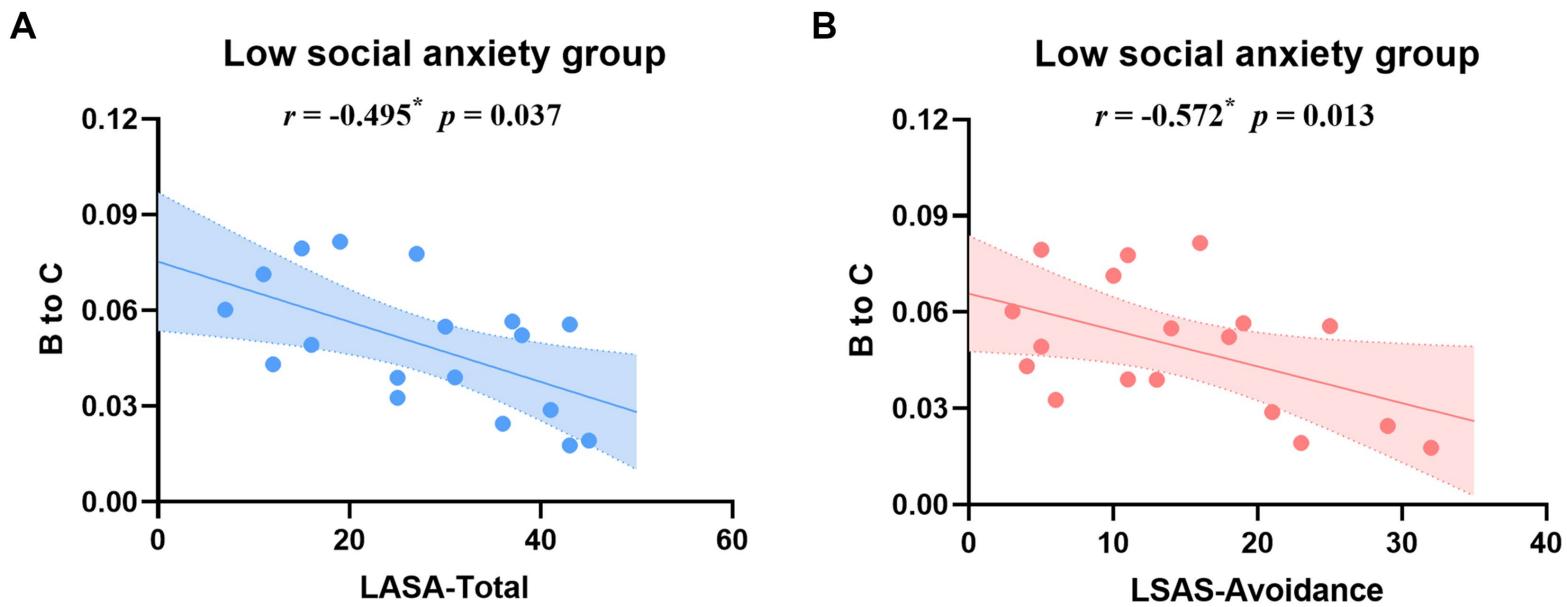
Correlation analysis between B → C transition probability and LSAS total scores **(A)** and LSAS-Avoidance subscale scores **(B)** in the low social anxiety group. Blue and pink lines represent linear regression fits with 95% confidence intervals (shaded areas). *p < 0.05.

The correlation analysis only revealed significantly negative correlations between B → C transition probability and both total LSAS scores (*r* = −0.572, *p* = 0.013) and LSAS-Avoidance scores (*r* = −0.495, *p* = 0.037) in the low social anxiety group (Figure 4). Detailed correlation coefficients are provided in Supplementary Tables S4, S5.

## 4 Discussion

This study explored the dynamic characteristics of brain functional networks in socially anxious individuals using EEG microstate analysis. The results showed that the high social anxiety group had longer duration and larger coverage of networks involved in processing personally significant information, self-reflection, and self-referential internal mentation (microstate C), while networks involved in executive functioning (microstate D) exhibited the opposite pattern. In terms of transition characteristics, the high social anxiety group showed significantly increased transition probabilities between networks associated with auditory/visual processing and arousal (microstate A) or self-visualization and autobiographical memory (microstate B) and networks processing personally significant information (microstate C) (A ↔ C, B ↔ C), and significantly decreased transition probabilities between auditory/visual processing networks and executive function networks (A ↔ D). Interestingly, in the low social anxiety group, the transition probability from self-visualization to personally significant information processing networks (B → C) was negatively correlated with both total social anxiety severity and avoidance symptoms, suggesting that the flexibility in transitioning between these networks might serve as a protective mechanism against social anxiety development. These pattern changes reflect enhanced processing of personally significant information with increased self-reflection (enhanced microstate C) and impaired executive functioning (weakened microstate D) in socially anxious individuals. Moreover, their state transition characteristics indicate a tendency to rapidly transform sensory and self-related visual information into personally significant information processing and self-referential internal mentation, providing new insights into understanding the neural mechanisms of social anxiety.

### 4.1 Temporal characteristics of microstates in social anxiety

The study found significant changes in microstate temporal characteristics in the high social anxiety group, showing increased presence of microstate C, which is associated with processing personally significant information and self-referential internal mentation, and decreased presence of microstate D, which is associated with executive functioning (Tarailis et al., 2024). These findings are consistent with previous fMRI studies on abnormal default mode network function in social anxiety. For example, Gentili et al. (2008) found abnormal default mode network function in patients with social anxiety disorder using resting-state fMRI.

This study forms a contrast and complement to previous anxiety-related research. Similar to the functional weakening of microstate D (reduced duration, occurrence, and coverage) observed in patients with generalized anxiety disorder (Hao et al., 2025), socially anxious individuals also exhibit functional weakening of microstate D. This suggests that different types of anxiety disorders may share certain characteristics of attentional network dysfunction. However, while GAD patients showed increased transitions between states C and E (Hao et al., 2025), and anxiety symptoms were associated with microstate E parameters and B to E transitions (Xue et al., 2024), the socially anxious individuals in this study mainly showed changes in microstates C and D. Unlike depressive symptoms which were found to positively correlate with the occurrence of microstate A (Damborská et al., 2019), our findings highlight distinct patterns in social anxiety. These differences in microstate characteristics may reflect the unique neural network dynamics associated with different types of emotional disorders, suggesting that specific anxiety subtypes may have their own characteristic patterns of brain network activity.

This study is the first to reveal the dynamic antagonistic relationship between the default mode network and attention network in social anxiety through microstate analysis. The discovery of these temporal dynamic characteristics extends the static functional connectivity findings of previous fMRI studies, providing a new dimension for understanding the neural mechanisms of social anxiety. In particular, the antagonistic relationship observed between microstates C and D provides new neurophysiological evidence for the excessive self-focused attention theory proposed by Clark and Wells (1995). This dynamic imbalance phenomenon supports Etkin and Wager's (2007) view of imbalanced attentional resource allocation in social anxiety. According to the cognitive model of Rapee and Heimberg (1997), this persistent self-focused state may enhance sensitivity to social threat cues.

### 4.2 Microstate transition characteristics in social anxiety

The social anxiety group exhibited a unique microstate transition pattern, mainly reflected in two aspects: enhanced transitions between sensory processing (microstates A and B) and personally significant information processing networks (microstate C), and weakened transitions between sensory processing and executive function networks (microstate D). These altered transition patterns suggest differences in the temporal organization of brain activity in individuals with high social anxiety traits. While these changes in network dynamics occur in parallel with known cognitive features of social anxiety, such as heightened self-focus and altered executive control, the causal relationships between specific transition patterns and cognitive processes require further investigation.

The study found that the high social anxiety group showed abnormal transition patterns between the sensory processing networks and networks involved in personally significant information processing. Specifically, the transition probabilities from networks associated with auditory/visual processing and arousal (microstate A) and self-related visual processing/autobiographical memory (microstate B) to networks involved in personally significant information processing and self-referential internal mentation (microstate C) were significantly increased. This pattern differs from that observed in GAD, where the primary abnormality involves increased transitions between personally significant information processing and interoceptive/emotional information processing networks (C ↔ E) (Hao et al., 2025), suggesting distinct network interaction patterns across anxiety subtypes. The increased transitions from sensory processing to personally significant information processing networks we observed may represent a neural basis for the predominant negative self-evaluation in social anxiety, as Dixon et al. (2022) found that while social anxiety disorder (SAD) patients and controls showed similar patterns of DMN recruitment, SAD patients distinctly endorsed significantly fewer positive traits and more negative traits during self-evaluation tasks. The enhanced transitions to networks involved in personally significant information processing are also consistent with the increased connectivity between the DMN and the amygdala and salience network reported in SAD patients (Lucherini Angeletti et al., 2023).

Simultaneously, we found that the high social anxiety group showed significantly weakened transitions related to networks involved in executive functioning (microstate D). This finding aligns with a broader pattern in anxiety disorders, as generalized anxiety disorder (GAD) patients exhibit reduced transition probabilities from all other states to microstate D (Hao et al., 2025), and similar weakened transitions involving executive function networks have been observed in emotional disorders more broadly (Al Zoubi et al., 2019). Given that microstate D is associated with executive functioning, these shared characteristics in transition patterns may reflect a common deficit in executive control among anxiety disorders, supporting existing evidence of attentional control deficits in social anxiety (Eysenck et al., 2007; Amir et al., 2009). However, our study revealed a distinct pattern specific to social anxiety: particularly weakened transitions between networks associated with auditory/visual processing and arousal (microstate A) and executive function networks (A ↔ D). This differs from both the pattern in GAD, where transitions to executive function networks are reduced from all states (Hao et al., 2025), and the pattern reported by Al Zoubi et al. (2019), where weakened transitions occurred primarily between networks involved in self-related visual processing and autobiographical memory (microstate B) and executive function networks (B ↔ D). This specificity may reflect the unique characteristics of social anxiety disorder, where individuals may have particular difficulties in dynamically coordinating sensory information processing (such as conversations and evaluations from others) with executive control processes.

Correlation analyses revealed that the transition probability from self-related visual processing to personally significant information processing networks (B → C) demonstrated significant negative correlations with both total LSAS scores and avoidance subscale scores exclusively in the low social anxiety group. Of particular significance is the specific association with avoidance symptoms, suggesting that these network transition patterns may be fundamentally linked to behavioral manifestations of social anxiety. In individuals with low social anxiety levels, this preserved flexibility in transitions between self-visual representation and personally significant information processing may constitute a neurophysiological protective mechanism, maintaining essential self-monitoring while preventing maladaptive self-referential processing. Conversely, the enhanced B → C transitions observed in the high social anxiety group may represent rigid, dysregulated processing patterns, aligning with previously documented negative self-evaluation bias (Dixon et al., 2022) and altered default mode network functioning (Lucherini Angeletti et al., 2023). These findings provide novel neurobiological evidence for understanding the protective and vulnerability factors in social anxiety, particularly regarding the neural mechanisms underlying avoidance behavior.

Uniquely, socially anxious individuals exhibit a systemic imbalance of enhanced transitions between sensory processing and personally significant information processing networks, and weakened transitions between sensory processing and executive function networks. This finding not only extends the existing understanding of the impact of anxiety on microstate dynamics but also provides a new perspective for understanding the neural mechanisms of social anxiety. These microstate dynamic characteristics have important implications for clinical interventions. First, the feature of enhanced transitions between sensory processing networks (microstates A and B) and networks involved in personally significant information processing (microstate C) indicates that clinical interventions need to focus on breaking individuals’ automated patterns of excessive self-focus. This can be achieved through executive control training or mindfulness interventions to enhance objective awareness of the external environment and reduce excessive processing of personally significant information (Wells, 2007). Second, the microstate pattern changes found in this study provide new ideas for biomarker research in social anxiety. These objective neural indicators can be used to assess treatment effects and provide quantitative feedback for clinical interventions. Furthermore, based on these findings, targeted neurofeedback training can be developed in the future. Existing research has shown that real-time regulation of prefrontal *α*-asymmetry can effectively reduce negative emotions and anxiety levels (Mennella et al., 2017), while amygdala neurofeedback training based on real-time functional magnetic resonance imaging can improve positive information processing in patients with depression (Young et al., 2017). This evidence supports the feasibility of improving symptoms by regulating the dynamic balance of brain networks through neurofeedback techniques, providing a theoretical basis for developing new intervention approaches.

### 4.3 Limitations and future directions

This study has several main limitations. First, we did not adequately control for common comorbid conditions such as generalized anxiety disorder, which may confound brain network dynamics (Brühl et al., 2014). Recent meta-analysis indicates that comorbidity indeed affects microstate patterns, particularly microstate B (Chivu et al., 2023). Future studies should strengthen the control of comorbid factors to better distinguish the neural characteristics specific to social anxiety. Second, we *a priori* selected four canonical microstate clusters for analysis rather than employing the more objective meta-criterion method. As Tarailis et al. (2024) pointed out, pre-setting the number of microstates may lead to misattribution of functional properties. Future studies could adopt this data-driven approach to determine the optimal number of microstates for a more comprehensive characterization of dynamic neural features in social anxiety. Additionally, the interpretation of microstate results requires careful consideration, as the relationship between microstates and functional networks is complex and not one-to-one (Michel and Koenig, 2018; Tarailis et al., 2024). Future multimodal studies combining EEG-fMRI and behavioral measures could help better establish the links between microstate dynamics and cognitive processes in social anxiety.

Despite these limitations, this study provides important insights into abnormal brain network dynamics in individuals with social anxiety. Combining microstate analysis with machine learning methods may identify novel biomarkers (Woo et al., 2017). Furthermore, developing real-time neurofeedback protocols targeting specific microstate parameters, especially those related to executive functioning (microstate D) and personally significant information processing (microstate C), could serve as an innovative therapeutic approach (Diaz Hernandez et al., 2016). These research directions will not only advance our understanding of the neural mechanisms underlying social anxiety but also promote progress in diagnosis and treatment methods.

## 5 Conclusion

This study revealed characteristic brain network dynamic patterns in socially anxious individuals. We found that these individuals exhibit significant microstate characteristics in the resting state: enhanced activity in networks related to processing personally significant information, self-reflection, and self-referential internal mentation (microstate C) and weakened executive functioning (microstate D). The study observed specific network transition patterns: frequent switching between networks associated with auditory/visual processing and arousal (microstate A) or self-visualization and autobiographical memory (microstate B) and networks involved in personally significant information processing and self-referential internal mentation (microstate C), accompanied by reduced engagement of executive function networks (microstate D). Importantly, in the low social anxiety group, the transition probability from self-related visual processing to personally significant information processing networks (B → C) showed significant negative correlations with social anxiety symptoms, particularly avoidance behaviors, suggesting that flexible network transitions might serve as a protective mechanism. This dynamic imbalance pattern reveals a key feature of social anxiety: even in the resting state, individuals struggle to break free from the repetitive cycle of transforming sensory and self-related visual information into personally significant information processing and self-referential internal mentation, while showing reduced executive functioning. These findings provide new insights into the neural mechanisms of social anxiety from a network dynamics perspective and offer directions for clinical intervention.

## Data Availability

The raw data supporting the conclusions of this article will be made available by the authors, without undue reservation.
