# Supplementary material for: Altered microstate C and D dynamics in high social anxiety: a resting-state EEG study
Source: Front Psychol. 2025 May 8;16:1581517. doi: 10.3389/fpsyg.2025.1581517 (PMC12095283; doi:10.3389/fpsyg.2025.1581517)
Supplement: Supplementary file 1 [file Supplementary_file_1.docx]

**Supplementary Table S1**

**Table 4-A.** Results of the correlation analysis between LSAS and its sub-dimensions and the four parameters of microstates in the **High Social Anxiety Group.**

| Relevance | | | |
| --- | --- | --- | --- |
|  | LSAS | LSAS-Fear/Anxiety | LSAS-Avoidance |
| Duration_A | 0.153 | 0.15 | 0.068 |
| Coverage_A | 0.185 | 0.103 | 0.151 |
| Density_A | 0.178 | 0.016 | 0.216 |
| Duration_B | -0.188 | 0.087 | -0.317 |
| Coverage_B | -0.205 | -0.012 | -0.255 |
| Density_B | -0.198 | -0.101 | -0.17 |
| Duration_C | -0.125 | -0.18 | -0.007 |
| Coverage_C | -0.079 | -0.214 | 0.081 |
| Density_C | 0.017 | -0.141 | 0.143 |
| Duration_D | 0.061 | 0.155 | -0.054 |
| Coverage_D | 0.065 | 0.146 | -0.041 |
| Density_D | 0.086 | 0.181 | -0.044 |

**Table Note:** Duration (ms): duration of microstates; Occurrence (/s): frequency of microstates per unit of time; Coverage (%): proportion of microstates covered.

**Table 4-B.** Results of correlation analysis between LSAS and its sub-dimensions and microstate transition probabilities in the **High Social Anxiety Group.**

| Relevance | | | |
| --- | --- | --- | --- |
|  | LSAS | LSAS-Fear/Anxiety | LSAS-Avoidance |
| A to B | -0.049 | -0.215 | 0.121 |
| A to C | 0.138 | -0.006 | 0.183 |
| A to D | 0.134 | 0.172 | 0.025 |
| B to A | 0.071 | 0.209 | -0.087 |
| B to C | -0.277 | -0.33 | -0.076 |
| B to D | -0.074 | 0.038 | -0.129 |
| C to A | 0.095 | -0.124 | 0.23 |
| C to B | -0.217 | -0.2 | -0.109 |
| C to D | 0.071 | 0.113 | -0.005 |
| D to A | 0.093 | 0.04 | 0.086 |
| D to B | -0.09 | 0.169 | -0.262 |
| D to C | 0.12 | 0.119 | 0.053 |

**Table Note:** A to B refers to the transition probability from microstate A to microstate B. The same applies to other expressions.

**Table 5-A.** Results of the correlation analysis between LSAS and its sub-dimensions and the four parameters of microstates in the **Low social anxiety group.**

| Relevance | | | |
| --- | --- | --- | --- |
|  | LSAS | LSAS-Fear/Anxiety | LSAS-Avoidance |
| Duration_A | -0.148 | 0.047 | -0.242 |
| Coverage_A | -0.203 | 0.027 | -0.303 |
| Density_A | -0.299 | -0.013 | -0.408 |
| Duration_B | 0.079 | 0.146 | 0.002 |
| Coverage_B | 0.07 | 0.173 | -0.032 |
| Density_B | -0.097 | 0.142 | -0.241 |
| Duration_C | -0.364 | -0.308 | -0.277 |
| Coverage_C | -0.426 | -0.376 | -0.313 |
| Density_C | -0.466 | -0.419 | -0.337 |
| Duration_D | 0.293 | 0.011 | 0.401 |
| Coverage_D | 0.212 | 0.007 | 0.291 |
| Density_D | -0.151 | -0.084 | -0.147 |

**Table Note:** Duration (ms): duration of microstates; Occurrence (/s): frequency of microstates per unit of time; Coverage (%): proportion of microstates covered.

**Table 5-B.** Results of correlation analysis between LSAS and its sub-dimensions and microstate transition probabilities in the **Low social anxiety group.**

| Relevance | | | |
| --- | --- | --- | --- |
|  | LSAS | LSAS-Fear/Anxiety | LSAS-Avoidance |
| A to B | 0.055 | 0.18 | -0.058 |
| A to C | -0.416 | -0.255 | -0.39 |
| A to D | -0.027 | 0.114 | -0.122 |
| B to A | 0.154 | 0.262 | 0.019 |
| **B to C** | **-.572*** | **-0.406** | **-.495*** |
| B to D | 0.219 | 0.24 | 0.126 |
| C to A | -0.423 | -0.225 | -0.422 |
| C to B | -0.43 | -0.256 | -0.409 |
| C to D | 0.061 | -0.185 | 0.223 |
| D to A | -0.19 | -0.091 | -0.198 |
| D to B | 0.303 | 0.314 | 0.188 |
| D to C | 0.106 | -0.146 | 0.257 |

**Table Note:** A to B refers to the transition probability from microstate A to microstate B. The same applies to other expressions; **p* < 0.05.
